# Development and effectiveness of a user-centered mobile application for informal caregivers of people living with dementia: randomized controlled trial

**DOI:** 10.1186/s12877-026-08235-7

**Published:** 2026-09-04

**Authors:** Bugse Yüceer, Fatma Ilknur Cinar, Mehmet Ilkin Naharci, Burcu Kadi

**Affiliations:** 1 Institute for Public Health and Nursing Research (IPP), Bremen University, Bremen, Germany; 2 Gülhane Faculty of Nursing, University of Health Sciences, Ankara, Türkiye; 3 Gülhane Faculty of Medicine & Gülhane Training and Research Hospital, Department of Geriatrics, University of Health Sciences, Ankara, Türkiye

**Keywords:** Caregivers, Caregiver burden, Dementia, Digital intervention, Mobile applications, Neuropsychiatric symptoms, Quality of life, User-centered design

## Abstract

**Background:**

Dementia places substantial physical, psychological, and social demands on informal caregivers, and caregiving needs may change across the dementia trajectory as cognitive, behavioral, and functional changes alter day-to-day care demands. Digital interventions may not fully address caregivers’ evolving and heterogeneous needs and preferences. This study aimed to develop a user-centered mobile application informed by caregiver-identified needs and preferences across the dementia trajectory and to evaluate its effectiveness.

**Methods:**

A randomized controlled trial was conducted following a qualitative intervention-development phase. The intervention was developed in accordance with the Medical Research Council framework for complex interventions and informed by principles of person-centered dementia care and qualitative interviews with informal caregivers. The application provided dementia education; communication and daily-care guidance organized to reflect changing caregiving needs; guidance for behavioral and psychological symptoms; caregiver self-care and stress-management resources; information on formal and informal support services; and customizable reminders. Caregivers were randomized to receive usual care with access to the mobile application for 8 weeks or usual care alone. Outcomes included caregiver burden (Zarit Burden Interview), quality of life (WHOQOL-BREF), neuropsychiatric symptoms (Neuropsychiatric Inventory), and caregiver distress related to these symptoms. System usability was assessed post-intervention in the intervention group. Outcomes were assessed at baseline and week 8 using repeated-measures analysis of variance.

**Results:**

A total of 86 caregivers were randomized, of whom 75 were included in the analysis (intervention, *n* = 37; control, *n* = 38). Significant group-by-time interactions were observed for overall quality of life (*p* < 0.001) and the psychological domain (*p* < 0.001), with greater improvements in the intervention group than in the control group. No significant group-by-time interactions were observed for caregiver burden, neuropsychiatric symptom severity, or caregiver distress. The application demonstrated good usability at week 8.

**Conclusions:**

The user-centered mobile application was associated with improvements in overall and psychological quality of life among participants included in the 8-week analysis, while no significant intervention effects were observed for caregiver burden, neuropsychiatric symptoms, or caregiver distress. Larger studies with longer follow-up and more detailed assessment of application engagement are needed to evaluate the durability and broader effects of the intervention.

**Trial registration:**

This trial was registered at ClinicalTrials.gov (Registration number: NCT06179667, Date of registration: 27 November 2023).

**Supplementary Information:**

The online version contains supplementary material available at https://doi.org/10.1186/s12877-026-08235-7.

## Background

Dementia is a chronic, progressive clinical syndrome characterized by decline in cognitive functions, such as memory, language, reasoning, and problem-solving, leading to increased dependence on activities of daily living [1]. With the global population aging, dementia has emerged as a major public health challenge [2]. According to the World Health Organization, more than 55 million people worldwide currently live with dementia, and this number is projected to rise to 78 million by 2030 and 139 million by 2050 [3]. Dementia is one of the leading causes of disability and dependency among older adults and represents a substantial burden for individuals, families, and health and social care systems [2].

As dementia progresses, affected individuals experience increasing difficulties in performing basic and instrumental activities of daily living, including eating, dressing, bathing, and toileting [3]. Changes in cognition and functional ability may also be accompanied by behavioral and psychological symptoms, such as agitation, depression, anxiety, sleep disturbances, hallucinations, personality changes, and wandering [4]. These changes may increase care needs and functional dependence, resulting in greater day-to-day caregiving demands [2, 3].

The majority of dementia care is delivered by informal caregivers, including family members, friends, and other non-professional individuals who provide unpaid support [5]. Informal caregivers play a central role in managing daily care tasks, coordinating medical appointments, monitoring symptoms, and providing emotional support [6]. While this role is essential for maintaining the well-being of people living with dementia (PLwD), it is often assumed without formal preparation or training. Consequently, caregivers are at an increased risk of experiencing adverse physical, psychological, social, and economic outcomes [7, 8]. Previous studies have consistently reported high levels of caregiver burden, stress, depressive symptoms, sleep disturbances, social isolation, and reduced quality of life among informal caregivers of PLwD [7, 9].

Caregiver burden and reduced quality of life are not only detrimental to caregivers but may also negatively affect the quality of care provided to PLwD [10]. Higher caregiver stress has also been associated with poorer caregiving outcomes and greater behavioral and psychological symptom burden in care recipients [8, 10]. Therefore, supporting informal caregivers is widely recognized as a key component of high-quality dementia care and a priority for public health interventions [2, 3].

Non-pharmacological interventions, including psychoeducation, skills training, psychosocial support, physical activity, mindfulness-based practices, and support groups, have demonstrated benefits for caregiver well-being and burden [7, 11]. However, access to such interventions is often limited by time constraints, caregiving responsibilities, geographical barriers and the availability of structured support services [12]. These barriers may become particularly important as care demands increase and caregivers have fewer opportunities to leave the PLwD unattended or participate in face-to-face programs [9].

In recent years, mobile health interventions have gained increasing attention as an accessible approach to support informal caregivers [13]. Mobile applications offer several advantages, including continuous availability, flexibility of use, and potential for personalization [14]. A growing number of mobile applications have been developed to support caregivers of PLwD by providing educational information, care-management tools, and resources aimed at supporting psychosocial well-being [15–17]. Some applications also incorporate cognitive or behavioral strategies intended to support caregiving skills and psychological well-being [18, 19]. However, digital interventions for dementia caregivers vary in their content, features, and degree of personalization and may not fully reflect caregivers’ heterogeneous needs and preferences [13–15, 19–21].

Caregiving demands may change across the dementia trajectory as cognitive, behavioral, and functional changes alter the level and type of assistance required in everyday care [6, 22]. Caregivers may also differ in their support needs and preferences, highlighting the importance of flexible and individualized support [14, 20]. Organizing caregiving guidance to reflect changing care demands across the dementia trajectory may increase the relevance of the support provided and enable caregivers to access information that is more closely aligned with their current care situations [13, 14]. A user-centered development approach is particularly relevant in this context because caregivers’ lived experiences, unmet needs and preferences can inform intervention content, organization and delivery [14, 20]. 

Accordingly, the mobile application developed in the present study was designed to respond to evolving caregiver needs across the dementia trajectory. It combined dementia education; practical guidance on communication and daily care organized to reflect changing caregiving needs; guidance for managing behavioral and psychological symptoms; caregiver self-care and stress-management resources; information on formal and informal support services; and customizable reminder functions. The present study aimed to develop this user-centered mobile application, informed by caregiver-identified needs and principles of person-centered dementia care and to evaluate its effectiveness in a randomized controlled trial.

### Study design and setting

This study comprised a qualitative intervention-development phase followed by a randomized controlled trial. The study was conducted between 2023 and 2025 at a tertiary geriatric clinic. Findings from the qualitative phase informed the content, organization, and design of the mobile application, while the randomized controlled trial evaluated its effectiveness. A fully functional Android-based mobile application was developed and used by intervention participants during the trial.

### Intervention development

The mobile application was developed in accordance with the Medical Research Council (MRC) framework for complex interventions. The framework was selected because the intervention comprised multiple interacting components, including educational content, practical caregiving guidance, psychosocial support, care-organization functions, and usability adaptations, and provided a structured approach to integrating evidence, stakeholder perspectives, contextual factors, and iterative refinement [23].

Principles of person-centered dementia care provided the conceptual basis for the caregiving content [23, 24]. These principles, including recognition of the individual’s values and identity, an individualized approach to care, consideration of the perspective of thePLwD, and promotion of a supportive social environment, informed recommendations on communication, daily care, and responses to behavioral and psychological symptoms [24].

Qualitative data were collected through in-depth, semi-structured interviews with informal caregivers of PLwD receiving follow-up care at a tertiary geriatric clinic. Eligible participants were adults aged ≥ 18 years who were the primary informal caregivers of a person diagnosed with major neurocognitive disorder according to DSM-5 criteria and who had provided care for at least one month for a minimum of six hours per day [15]. Formal caregivers and individuals with self-reported neurological or psychiatric conditions were excluded. The interviews were conducted to identify caregivers’ experiences, caregiving challenges, unmet needs, and preferences for digital support and thereby inform the user-centered development of the intervention. Purposive sampling was used to include caregivers supporting PLwD with differing levels of cognitive and functional impairment. Interviews were conducted in a private room at the geriatric clinic, lasted approximately 40–60 min, and were undertaken by the first author, who had formal training and teaching experience in qualitative research methods. Interviews were audio-recorded following written informed consent and analyzed using Colaizzi’s phenomenological method with MAXQDA 22 software [25]. Seventeen caregivers participated, and data saturation was achieved.

The qualitative analysis identified three broad areas that directly informed intervention development: (1) cognitive, behavioral, and functional changes experienced by PLwD; (2) psychological, social, physical, and economic consequences of caregiving; and (3) caregiver needs related to dementia information, management of dementia-related symptoms, psychological and social support, and access to community and professional services. These findings informed the application’s three main content modules: dementia-related education, dementia management and daily-care support, and caregiver support and self-care. They also informed the reminder and care-organization functions. For intervention-development purposes, caregiver-identified needs and related qualitative findings were synthesized according to their relevance to application content, organization, and functions. Their mapping to application responses and intended outcomes is presented in Table 1.

**Table 1:** Mapping caregiver-identified findings to mobile application components

Caregiver-identified finding	Relevance across the dementia trajectory and individual context	Application response	Intended outcome
Limited understanding of dementia and its progression; differing preferences regarding information about what to expect as dementia progresses	Some caregivers wanted information about the entire dementia course from the outset, whereas others preferred information relevant to current caregiving needs	Dementia education covering progression, stages, symptoms, and treatment; user-selectable sections allowed caregivers to access information according to their information needs	Improve access to understandable and relevant dementia-related information
Difficulties with communication and practical daily-care activities	Early- and moderate-dementia caregiving challenges showed substantial overlap, whereas increasing functional dependence was associated with greater hands-on care needs	Separate early–moderate and advanced guidance for communication, nutrition, dressing, bathing, mobility and transfers, safety, and other daily-care activities; caregivers selected the section most relevant to current care needs	Support management of changing daily-care demands
Challenges in responding to behavioral and psychological symptoms	Could arise across the dementia trajectory and varied according to the individual and presenting problem	Problem-based guidance for common behavioral and psychological symptoms, including hallucinations, repetitive behaviors, sleep problems, and wandering; scenario-based guidance for selected situations	Support caregivers in responding to behavioral and psychological symptoms
Psychological distress, stress, fatigue, and self-care difficulties	Could occur throughout caregiving and vary in nature, intensity, and priority	Stress management, cognitive reframing, physical activity, nutrition, sleep, mindfulness, and customizable breathing exercises	Support caregiver self-care, psychological well-being, and quality of life
Social isolation and need for social, professional, community, and financial support	Type and priority of support varied according to individual circumstances and over time	Information on family and social support, home-care services, residential care, crisis support, disability-related rights, and financial support	Facilitate access to appropriate formal and informal support resources
Difficulties organizing daily caregiving activities	Organizational needs varied according to individual care routines and caregiving demands	Customizable reminders for medications, appointments, toileting, hydration, walking, and other daily-care activities	Support planning and organization of daily care
Preference for simple, accessible, individualized application use	Preferences for accessing and using digital support varied among caregivers	Simplified navigation and visual presentation, user-selectable content, home-screen information, profile/privacy information, and frequently asked questions	Support accessible and independent application use

The Medical Research Council framework guided intervention development and evaluation, while principles of person-centered dementia care informed the caregiving content. Intended outcomes represent intervention targets rather than demonstrated component-specific effects

Caregiver needs varied across the dementia trajectory and also overlapped across individuals and care situations. The clearest variation concerned practical caregiving demands associated with increasing functional dependence. Because practical challenges reported in early and moderate dementia substantially overlapped, related guidance was grouped within an early–moderate section, whereas advanced-dementia guidance addressed greater hands-on assistance associated with increasing functional dependence. This represented a flexible way of organizing practical-care content rather than fixed or mutually exclusive categories of caregiver need.

The qualitative findings also indicated that psychological distress, self-care difficulties, and needs for social, professional, and community support could arise throughout caregiving and vary in nature, intensity, and priority over time. Accordingly, psychosocial, self-care, and support resources were not linked to a specific dementia grouping. Similarly, behavioral and psychological symptom-management content was organized around the presenting caregiving problem rather than by dementia stage.

Caregivers differed in their preferences for information about symptoms and care needs that may arise as dementia progresses. Some wished to know in advance what they might encounter in later stages, whereas others indicated that receiving information about later-stage symptoms could increase anxiety and preferred to focus on their current caregiving needs. Accordingly, content was organized into separate, user-selectable sections so that caregivers could access information according to their individual needs and preferences.

The qualitative findings were translated into application content and functions through an iterative multidisciplinary development process involving a geriatrician, a clinical nurse, a social worker, a psychological counselor, a computer engineer, academic researchers with expertise in dementia care, and informal caregivers. Caregiver-identified needs were considered alongside current evidence on dementia care and caregiver support [4, 7, 11, 24]. Clinical and psychosocial input informed the relevance and practical applicability of dementia-care and caregiver-support content, while technical input informed the feasibility, organization, navigation, and implementation of application functions. Feedback from the multidisciplinary development team led to the addition of crisis-related contact information and a frequently asked questions section and further refinement of formal and informal support resources and reminder functions.

Caregiver involvement continued during prototype refinement. Three caregivers from the qualitative phase contributed to this stage and did not participate in the RCT. Their feedback on relevance, comprehensibility, accessibility, and ease of use informed further refinement of the application. Written and video content was refined, language and navigation were simplified, and visual presentation was adjusted. The home screen was modified to display recent reminders and application-use information. Individualized functions were also refined; for example, caregivers could select and save their preferred inhalation, breath-holding, and exhalation durations for subsequent breathing exercises.

As part of the final stage of intervention development, the application was evaluated by six experts with expertise spanning geriatric medicine, internal medicine nursing, geriatric care, home-care nursing, and computer engineering. Experts were provided electronically with access to the application and its written content. Information quality and reliability were assessed using the DISCERN instrument, yielding a mean score of 73.83 ± 8.70. Application usability was assessed using the Mobile Application Usability Scale, with a mean score of 252.5 ± 23.96.

In addition to the standardized assessments, expert recommendations addressed written expression and clarity, content organization and presentation, and visual and interface-related features. The research team reviewed these recommendations before finalizing the intervention. Where indicated, wording and content presentation were revised to improve clarity, comprehensibility, and consistency, and relevant visual and interface elements were adjusted to improve accessibility and ease of use. These refinements were completed before implementation of the RCT.

### Participants and sample size

The study population consisted of informal caregivers of PLwD who were providing care to individuals receiving routine follow-up at the geriatric clinic. Participants were eligible if they were aged 18 years or older; were able to read and write in Turkish; were the primary informal caregiver of a person diagnosed with major neurocognitive disorder according to the DSM-5 criteria; had been providing care for at least one month for a minimum of six hours per day [15, 26]; and owned an internet-enabled Android smartphone with the ability to use mobile applications independently.

Individuals were excluded if they were formal (paid) caregivers or had a history of neurological or psychiatric disorders. Intervention-group participants who did not access the application at least once per week during the 8-week intervention were discontinued from the intervention in accordance with the prespecified protocol.

Sample size calculation was based on caregiver burden outcomes reported in a comparable intervention study by Ferré-Grau et al., which demonstrated a moderate-to-large effect size (Cohen’s d = 0.68) [27]. Using an alpha level of 0.05 and a power of 80%, calculations performed with G*Power version 3.1.9.4 indicated that a minimum of 37 participants per group was required. Allowing for a potential attrition rate of 15%, the target sample size was increased to 43 participants per group, yielding a total sample of 86 caregivers [28].

### Randomization and blinding

Stratified block randomization was used to improve balance between groups with respect to caregiver age and dementia stage of the care recipient, both of which have been associated with caregiver burden [6, 22]. Caregivers were stratified by age (≤ 45 vs ≥ 46 years) and care recipients by dementia stage (early–moderate vs advanced). Dementia stage was obtained from the care recipient’s medical record. The age threshold was prespecified for randomization purposes and was not intended to represent a clinically validated cutoff. Because few care recipients had early-stage dementia, early and moderate dementia were combined into a single stratum. Within strata, participants were randomized in a 1:1 ratio using the Sealed Envelope web-based system with randomly varying block sizes of four and six. Allocation was performed by an independent researcher who was not involved in recruitment or data collection. Because of the nature of the intervention, participants and researchers involved in intervention delivery could not be blinded to group allocation. Statistical analyses were conducted by a researcher blinded to group allocation.

### Study procedures

The study was conducted over an 8-week period. Data were collected at two time points: baseline and post-intervention at week 8. Baseline assessments included sociodemographic and caregiving characteristics, caregiver burden, quality of life, and neuropsychiatric symptoms of PLwD. Follow-up assessments at week 8 included caregiver burden, quality of life, and neuropsychiatric symptoms using the same instruments. In addition, usability of the mobile application was assessed post-intervention in the intervention group using the System Usability Scale.

Baseline and follow-up data were collected electronically using a standardized online questionnaire. Participants received survey links electronically through a secure messaging platform (WhatsApp), and electronic informed consent was obtained prior to participation. Participants received no financial or material compensation for participation. The trial is reported in accordance with the CONSORT 2025 statement, with relevant additional guidance from the CONSORT extension for randomized trials of nonpharmacologic treatments [29].

### Intervention

A fully functional Android-based mobile application was used in the intervention. Participants allocated to the intervention group continued to receive usual care and additionally received access to the application for 8 weeks. The application comprised three main content modules:

1. Dementia-related education: This module provided structured information on dementia, including its definition, types, risk factors, diagnosis, progression and stages, symptoms, and treatment options. The content was designed to provide caregivers with accessible information about dementia and its course.
2. Dementia management and daily-care support: This module provided practical guidance on person-centered dementia care, communication, nutrition, dressing, bathing, toileting, mobility and transfers, safety-related issues, shopping, meal preparation, financial activities, and medication management. For each practical daily-care topic, separate early–moderate and advanced sections were provided. Early–moderate guidance generally focused on maintaining independence, establishing routines, using reminders and step-by-step prompting, and adapting the environment, whereas advanced-dementia guidance addressed greater hands-on assistance, safety, comfort, and care needs associated with increasing functional dependence. For example, nutrition guidance included routines, reminders, and supported independence in the early–moderate section, whereas the advanced section addressed feeding assistance and chewing and swallowing difficulties. Caregivers could select and access the section most relevant to the care recipient’s current needs.

The module also included problem-specific guidance on memory problems and common behavioral and psychological symptoms, including hallucinations and delusions, repetitive behaviors, sleep problems, aggression, wandering and getting lost, depression, anxiety, and apathy. Scenario-based instructional videos accompanied selected topics to demonstrate practical approaches to situations caregivers may encounter in daily care.

(3) Caregiver support and self-care: All caregiver support and self-care resources were available regardless of which practical-care section caregivers used. This module included information on informal and formal support resources, including family and social support, support groups, home-care services, day-care services, residential care, psychological counseling, crisis-related support, disability-related rights, and financial support. Self-care content addressed stress management, cognitive reframing, physical activity, nutrition, sleep, hobbies, and personal time. Mindfulness practices and breathing exercises were provided through video and audio materials. The breathing-exercise function allowed users to select and save their preferred inhalation, breath-holding, and exhalation durations for subsequent use.

In addition to the three main content modules, the application included a reminder function through which caregivers could schedule medications, appointments, toileting, hydration, cognitive activities, walking, and other daily-care activities. The home screen displayed current reminders and application-use information. The application also included profile, privacy and data-security, password-management, and frequently asked questions functions. Representative screenshots of the application interface and key functions are provided in Supplementary File 1.

Following baseline assessment, intervention participants received individualized login credentials and were instructed not to share their account information. A brief instructional video was provided to support initial familiarization with the application, and technical assistance was available when needed. Application use was otherwise unrestricted, allowing caregivers to access content as frequently as they wished according to their needs.

At least one application access per week was prespecified as the minimum use criterion for continued participation. This criterion provided an operational definition of ongoing engagement with the self-directed intervention and was not intended to represent an established therapeutic dose. Weekly telephone calls were limited to monitoring application use and addressing technical or access-related difficulties; no caregiving counseling or additional intervention content was provided during these calls. Participants who did not meet this criterion were discontinued from the intervention in accordance with the prespecified protocol.

A secure researcher dashboard was used to monitor application use during the intervention. The dashboard recorded cumulative application use, average session duration, and the date of most recent activity. The most recent activity date was reviewed weekly to determine whether the prespecified minimum weekly application-use criterion had been met.

### Control group

Participants allocated to the control group continued to receive routine clinical care at the geriatric clinic and did not receive access to the mobile application or any additional structured digital caregiver intervention during the 8-week study period. Usual care was based on comprehensive geriatric assessment and multidisciplinary follow-up; according to individual clinical needs and family requests, caregivers could also receive routine information and counseling on dementia progression, communication, home care, behavioral symptoms and available psychosocial support. The intervention group continued to receive the same usual care, with access to the mobile application constituting the additional intervention component. Following completion of study assessments, control-group participants were offered access to the application.

### Outcome measures

A researcher-developed questionnaire was used to collect sociodemographic and caregiving-related information based on literature. The form consisted of 23 items, including eight questions on caregivers’ sociodemographic characteristics (e.g., age, gender, education level, marital status, income status) and fifteen questions on caregiving characteristics (e.g., duration of caregiving, daily caregiving hours, gender of PLwD) [15, 20, 26, 30]. The questionnaire was developed to collect factual sociodemographic and caregiving information rather than to measure a psychometric construct; therefore, internal-consistency reliability was not applicable. No formal psychometric validation was undertaken.

Caregiver burden was assessed using the Zarit Burden Interview (ZBI) [31, 32]. The 22-item ZBI assesses perceived burden associated with caregiving. Items are rated from 0 (never) to 4 (nearly always), yielding a total score from 0 to 88, with higher scores indicating greater caregiver burden [31]. The Turkish version has demonstrated validity and reliability [32].

Caregiver quality of life was measured using the World Health Organization Quality of Life Scale Brief Version (WHOQOL-BREF), developed by the World Health Organization to assess quality of life across physical, psychological, social, and environmental domains [33]. The Turkish version includes an additional culturally adapted environmental item, resulting in a total of 27 items [34]. Items are rated on a 5-point Likert scale, with higher domain scores indicating better perceived quality of life [34].

Neuropsychiatric symptoms of PLwD were assessed using the Neuropsychiatric Inventory (NPI), which evaluates behavioral and psychological symptoms across 12 domains, including delusions, hallucinations, agitation, depression, anxiety, apathy, irritability, and sleep and appetite disturbances [35, 36]. The inventory is completed by the informal caregiver, who reports the presence, frequency, and severity of symptoms observed in the PLwD. Total scores range from 12 to 144, with higher scores indicating greater neuropsychiatric symptom burden [36].

Usability of the mobile application was evaluated post-intervention using the System Usability Scale (SUS), a standardized tool for assessing the usability of digital systems [37, 38]. The scale consists of 10 items rated on a 5-point Likert scale and yields a total score ranging from 0 to 100, with higher scores indicating better usability [37].

### Statistical analysis

Statistical analyses were conducted using SPSS version 29.0. Normality assumptions were assessed using skewness and kurtosis values, and homogeneity of variances was examined using Levene’s test. Continuous variables were represented as means and standard deviations, whereas categorical variables were reported as frequencies and percentages. Baseline comparisons between groups were performed using independent samples t-tests or Pearson’s chi-square tests. Variables not meeting normality assumptions were analyzed using Mann–Whitney U tests. The effects of time, group, and group-by-time interaction were analyzed using two-way repeated measures analysis of variance. The statistical significance threshold for the two‐tailed test and the analyses was set at *p* < 0.05.

The efficacy analyses were conducted using a per-protocol complete-case population comprising participants who completed the post-intervention assessment and, for those in the intervention group, met the prespecified minimum weekly application-use criterion. Participants without post-intervention outcome data were not included in the analyses, and no imputation of missing outcome data was performed.

### Ethical considerations

Ethical approval was obtained from the Health Science University Human Research Ethics Committee (approval number: E-50687469-799-218688179). The study was conducted in accordance with the Declaration of Helsinki. The trial was registered at ClinicalTrials.gov (NCT06179667). Electronic informed consent was obtained from all participants prior to enrollment. Participants were informed that they could withdraw at any time without providing a reason and without any effect on their clinical care.

## Results

### Participant flow

**Fig. 1:**
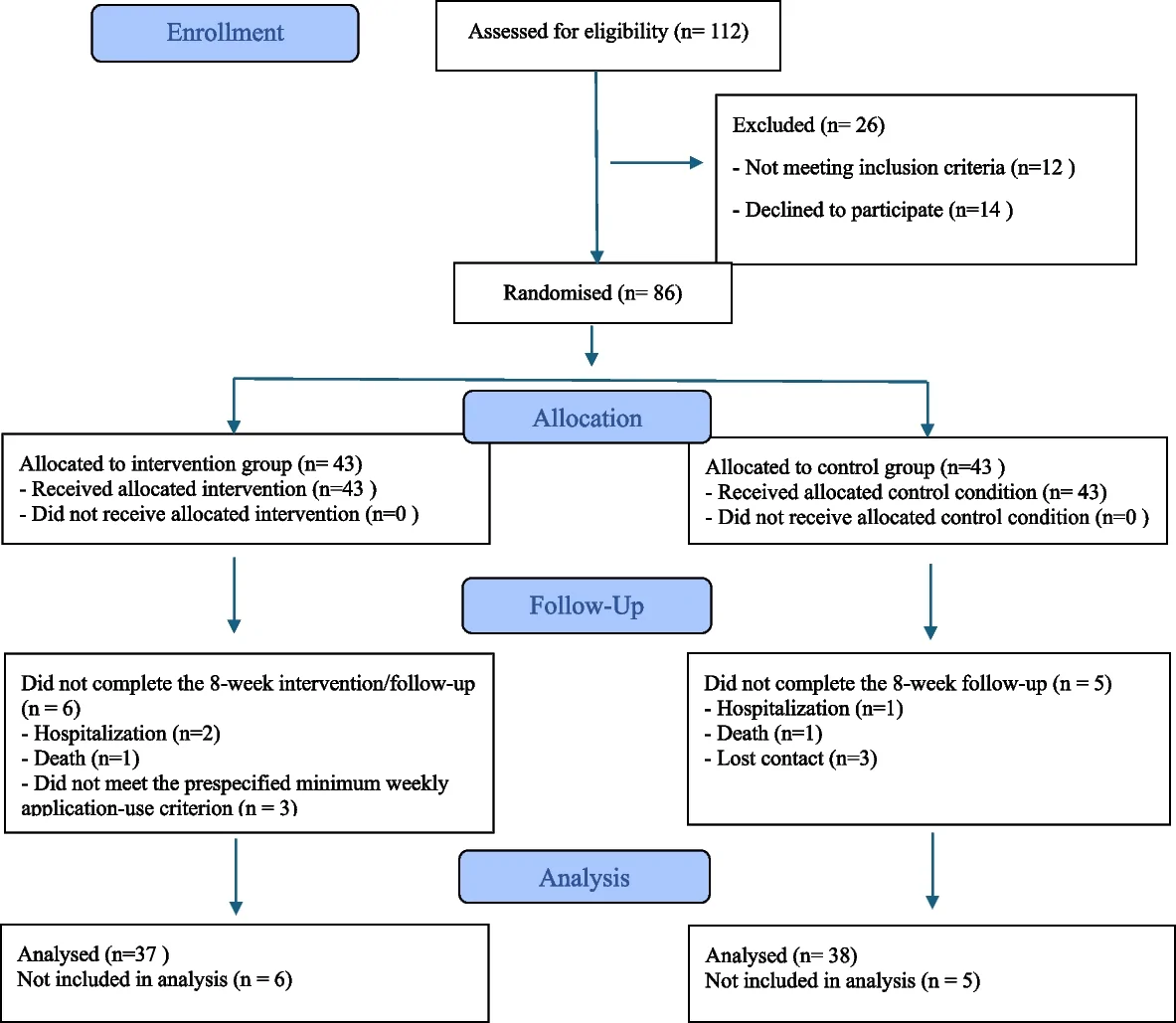
CONSORT 2025 flow diagram

A total of 86 caregivers were randomized, with 43 allocated to the intervention group and 43 to the control group. Six participants in the intervention group discontinued before post-intervention assessment because of hospitalization (*n* = 2), death of the care recipient (*n* = 1), or failure to meet the prespecified minimum application-use criterion (*n* = 3). Five participants in the control group discontinued because of hospitalization (*n* = 1), death of the care recipient (*n* = 1), or loss to follow-up (*n* = 3). Accordingly, 75 participants were included in the per-protocol complete-case analysis (intervention, *n* = 37; control, *n* = 38). Participant flow and reasons for discontinuation are presented in Fig. 1.

### Characteristics of participants

Table 2 presents the comparison of baseline characteristics of informal caregivers in the intervention and control groups. No statistically significant differences were observed between the groups with respect to age, gender, marital status, education level, income status, employment status, relationship to PLwD, presence of chronic disease, prior dementia-related education, or use of community dementia services (all *p* > 0.05).

**Table 2:** Characteristics of informal caregivers by study group (*n* = 75)

Characteristics	**Intervention Group (*n* = 37)** **n (%)**	**Control Group (*n* = 38)** **n (%)**	**Test statistics**
Age (years) (mean ± SD)	50.81 ± 8.98	51.42 ± 8.55	*t* = *−0.301* *p* = *0.437*
Gender			
Female	26 (70.27)	24 (63.16)	*χ*^*2*^* 0.427* *p* = *0.514*
Male	11 (29.73)	14 (36.84)	
Marital status			
Married	26 (70.27)	29 (76.32)	*χ*^*2*^ = *0.350* *p* = *0.554*
Single	11 (29.73)	9 (23.68)	
Educational level			
Literate/primary school	6 (16.22)	11 (28.95)	*χ*^*2*^ = *1.823* *p* = *0.402*
Secondary school	15 (40.54)	12 (31.58)	
University or higher	16 (43.24)	15 (39.47)	
Income status			
Income < expenses	5 (13.51)	9 (23.68)	*χ*^*2*^ = *1.948* *p* = *0.378*
Income = expenses	25 (67.57)	25 (65.79)	
Income > expenses	7 (18.92)	4 (10.53)	
Employment status			
Employed	16 (43.24)	18 (47.37)	*χ*^*2*^ = *0.129* *p* = *0.720*
Unemployed	21 (56.76)	20 (52.63)	
Relationship to the person with dementia			
Spouse	2 (5.40)	4 (10.53)	*Not evaluated*
Child	29 (78.38)	29 (76.32)	
Child-in-law	4 (10.82)	4 (10.53)	
Grandchild	2 (5.40)	1 (2.62)	
Presence of chronic disease			
Yes	13 (35.14)	14 (36.84)	*χ*^*2*^ = *0.024* *p* = *0.878*
No	24 (64.86)	24 (63.16)	
Previous dementia-related education			
Yes	15 (40.54)	15 (39.47)	*χ*^*2*^ = *0.009* *p* = *0.925*
No	22 (59.46)	23 (60.53)	
Use of dementia-related community services			
Yes	6 (16.22)	5 (13.16)	*χ*^*2*^ = *0.140* *p* = *0.708*
No	31 (83.78)	33 (86.84)	

*P*-value < 0.05 was considered statistically significant; Statistical analysis was not performed for variables with expected cell counts < 5

*SD* standard deviation, *t* independent samples t-test, *χ*^*2*^ Pearson’s chi-square test

Caregiving-related characteristics of informal caregivers are shown in Table 3. The mean age of PLwD was 81.62 ± 6.43 years in the intervention group and 79.84 ± 5.12 years in the control group, with ages ranging from 66 to 96 years and 67 to 91 years, respectively. The intervention and control groups did not differ significantly in terms of duration of caregiving, daily caregiving hours, age of the PLwD, number of individuals under care, gender of the care recipient, dementia stage, or dementia type (all *p* > 0.05).

**Table 3:** Caregiving-related characteristics by study group (*n* = 75)

Characteristics	Intervention Group (*n* = 37)	Control Group (*n* = 38)	Test statistics
Duration of caregiving, years (mean ± SD)	5.23 ± 0.98	4.31 ± 0.54	*t* = *0.819* *p* = *0.349*
Daily caregiving time, hours, median (Q1–Q3)	9 (7–24)	12 (7.75–24)	*U* = *465.500* *p* = *0.670*
Age of person with dementia, years (mean ± SD)	81.62 ± 6.43	79.84 ± 5.12	*t* = *1.327* *p* = *0.518*
Number of other dependents cared for (mean ± SD)	2.02 ± 0.89	2.34 ± 0.70	*t* = *−1.691* *p* = *0.805*
Gender of person with dementia, n (%)			
Female	24 (64.86)	22 (57.89)	*χ*^*2*^ = *0.384*
Male	13 (35.14)	16 (42.11)	*p* = *0.535*
Stage of dementia, n (%)			
Early/middle stage	25 (67.57)	25 (65.79)	*χ*^*2*^ = *0.027* *p* = *0.870*
Advanced stage	12 (32.43)	13 (34.21)	
Type of dementia, n (%)			
Alzheimer’s disease	25 (67.57)	27 (71.05)	*Not evaluated*
Vascular dementia	8 (21.63)	6 (15.79)	
Frontotemporal dementia	2 (5.40)	4 (10.53)	
Lewy body dementia	2 (5.40)	0	
Parkinson’s disease dementia	0	1 (2.63)	

*P*-value < 0.05 was considered statistically significant; Statistical analysis was not performed for variables with expected cell counts < 5

*SD* standard deviation, *Q1* 25th percentile, *Q3* 75th percentile, *t* independent samples t-test, *U* Mann–Whitney U test; *χ*^*2*^ Pearson’s chi-square test

### Intervention effects on caregiver outcomes

Changes in ZBI, WHOQOL-BREF, and NPI scores over time are presented in Table 4. For caregiver burden, no statistically significant group-by-time interaction was observed for the total ZBI score (*F* = 3.578, *p* = 0.063, η^2^ = 0.047). For overall quality of life, measured using the WHOQOL-BREF total score, a statistically significant group-by-time interaction was found (*F* = 14.247, *p* < 0.001, η^2^ = 0.163). Analysis of the WHOQOL-BREF subdomains showed a significant group-by-time interaction for the psychological domain (*F* = 22.566, *p* < 0.001, η^2^ = 0.236). No statistically significant group-by-time interactions were observed for the physical health, social relationships, or environmental domains (all *p* > 0.05). For NPI total scores, there was no significant main effect of time (*F* = 0.355, *p* = 0.553). A significant main effect of group was observed (*F* = 7.688, *p* = 0.007), whereas the group-by-time interaction was not significant (*F* = 1.791, *p* = 0.185, η^2^ = 0.024). No statistically significant differences were found for NPI distress scores with respect to group, time, or group-by-time interaction (all *p* > 0.05).

**Table 4:** Changes in ZBI, WHOQOL-BREF, and NPI scores over time between intervention and control groups (*n* = 75)

Outcome	Group IG (*n* = 37) CG (*n* = 38)	Pre-intervention, mean ± SD	Post-intervention, mean ± SD	Group (F; p)	Time (F; p)	Group × Time (F; p; η^2^)
ZBI (total)	IG	41.78 ± 13.93	38.97 ± 12.12	0.749; 0.390	2.552; 0.114	3.578; 0.063; 0.047
	CG	42.97 ± 16.20	43.21 ± 41.12			
WHOQOL-BREF (total)	IG	85.37 ± 11.59	88.59 ± 10.41	5.159; **0.026**	0.104; 0.748	14.247; ** < 0.001;** 0.163
	CG	82.86 ± 12.50	80.15 ± 9.08			
– Physical	IG	23.13 ± 4.55	23.00 ± 4.23	0.269; 0.605	0.089; 0.766	0.017; 0.896; 0.000
	CG	23.55 ± 4.15	23.50 ± 3.185			
– Psychological	IG	19.45 ± 3.17	22.29 ± 3.96	17.402; ** < 0.001**	5.629; **0.020**	22.566; ** < 0.001**; 0.236
	CG	18.50 ± 3.29	17.55 ± 3.21			
– Social relationships	IG	8.54 ± 2.12	8.73 ± 2.11	0.048; 0.827	0.085; 0.772	2.241; 0.139; 0.030
	CG	8.73 ± 2.11	8.50 ± 2.07			
– Environment	IG	28.43 ± 4.28	28.43 ± 4.37	9.760; **0.003**	3.775; 0.056	3.775; 0.056; 0.049
	CG	26.47 ± 4.44	24.65 ± 4.16			
NPI total score	IG	36.24 ± 18.99	34.45 ± 16.12	7.688; **0.007**	0.355; 0.553	1.79; 0.185; 0.024
	CG	24.52 ± 18.61	25.21 ± 13.03			
NPI distress score	IG	15.37 ± 7.49	14.32 ± 5.43	2.588, 0.112	0.940, 0.335	1.554, 0.217, 0.021
	CG	12.71 ± 5.90	12.84 ± 4.64			

*P*-value < 0.05 was considered statistically significant

*IG* Intervention Group, *CG* Control Group, *ZBI* Zarit Burden Interview, *WHOQOL-BREF* World Health Organization Quality of Life Scale Brief Version, *NPI* Neuropsychiatric Inventory, *SD* standard deviation, *F* two-way repeated measures analysis of variance (ANOVA), *η*^*2*^ effect size (eta squared)

### System usability of the mobile application

System usability outcomes for the mobile application are presented in Table 5. The mean SUS score at the end of the intervention (week 8) in the intervention group was 74.79 ± 15.97.

**Table 5:** Post-intervention SUS scores of the intervention group (*n* = 37)

Measure	Mean ± SD
System Usability Scale^*^	74.79 ± 15.97

*SUS* System Usability Scale, *SD* standard deviation

^*^Total scale ranges between 0 and 100

### Application use and adherence

Three of the 43 participants allocated to the intervention group (7.0%) did not meet the prespecified minimum weekly application-use criterion and were therefore not included in the per-protocol analysis. Among the 37 intervention participants included in the analysis, median cumulative application use during the 8-week intervention was 72 min (IQR 26–184; range 3–1508), and median average session duration was 10 min (IQR 6–21; range 1–188). The date of most recent activity was used for weekly adherence monitoring.

## Discussion

This randomized controlled trial evaluated a user-centered mobile application designed to address caregiver-identified needs across the dementia trajectory. Over the 8-week intervention period, the application was associated with improvement in caregivers’ overall quality of life and psychological quality of life. In contrast, no significant intervention effects were observed for caregiver burden, neuropsychiatric symptoms of PLwD, or caregiver distress related to these symptoms.

The improvement in quality-of-life outcomes is broadly consistent with evidence that digital interventions can support aspects of caregiver well-being [17, 21, 39, 40], although previous studies have reported heterogeneous findings [41, 42]. Differences across studies may partly reflect variation in intervention content, duration, and delivery [39, 42]. The significant psychological-domain finding may be relevant to the caregiver-focused components of the application, which included stress-management information, mindfulness, breathing exercises, and self-care resources. However, the study was not designed to evaluate component-specific effects or mediating mechanisms, and the observed quality-of-life changes cannot therefore be attributed to any particular application feature.

The intervention was developed through a structured, user-centered process in which the MRC framework guided development and evaluation, principles of person-centered dementia care informed the caregiving content, and qualitative caregiver findings shaped the organization of practical guidance and support resources [23, 24]. This approach allowed the intervention content to reflect variation in caregiver needs and preferences across the dementia trajectory.

Caregiver burden did not show a significant group-by-time effect. Previous digital interventions for informal caregivers of PLwD have similarly reported mixed findings, with some studies demonstrating reductions in burden [15, 19, 27] and others finding no significant effects [17, 42–44]. Caregiver burden is a multidimensional construct associated with caregiver, care-recipient, and contextual factors, including caregiving demands, functional dependence, behavioral and psychological symptoms, and available support [22, 45, 46]. In the present study, improvement in psychological quality of life was not accompanied by a significant reduction in caregiver burden. The relationship between changes in these outcomes cannot be determined from the present data and warrants investigation in future longitudinal studies.

No significant group-by-time effects were observed for neuropsychiatric symptom severity or caregiver distress related to these symptoms. Behavioral and psychological symptoms are influenced by multiple clinical, environmental, and contextual factors [16, 47]. Although the application provided caregivers with practical guidance for responding to these symptoms, it was a caregiver-support intervention rather than a clinical treatment for neuropsychiatric symptoms. Because these symptoms are multifactorial, changes in these outcomes may depend on clinical and contextual factors not directly addressed by the intervention [47–49].

The duration and intensity of intervention exposure should also be considered when interpreting the nonsignificant outcomes. Digital interventions for dementia caregivers vary considerably in duration and content, and studies have reported substantial variation in intervention engagement and its associations with caregiver outcomes [50–52]. Application use in the present study was otherwise unrestricted, although the protocol specified at least one application access per week as the minimum use criterion, and cumulative use and average session duration varied across participants. Whether greater or more sustained application use would have resulted in changes in caregiver burden, neuropsychiatric symptoms, or caregiver distress cannot be determined from the present study. The available application-use metrics provided only a broad characterization of intervention exposure. Future studies should prospectively collect more granular application-use data and examine potential associations between the frequency, duration, and type of engagement and intervention outcomes.

The application demonstrated good system usability, with a mean SUS score of 74.79 ± 15.97 [53]. This finding indicates that the application was generally perceived as usable by intervention participants who completed the study. Usability is an important consideration in digital caregiver interventions because difficulties with navigation, presentation, or accessibility may hinder continued use [13, 14, 53]. Longer-term evaluations should consider application use and engagement alongside digital literacy, accessibility, and contextual factors that may influence continued use.

### Limitations

Several limitations should be considered when interpreting the findings. First, the relatively modest sample size may have limited the precision of the effect estimates and the ability to detect smaller intervention effects. Recruitment from a single tertiary geriatric clinic may also limit the generalizability of the findings to caregivers in other clinical, community, and sociocultural settings.

Second, the analyses were based on a per-protocol complete-case population. Post-randomization discontinuations, including the exclusion of three intervention participants who did not meet the prespecified minimum application-use criterion, may have introduced attrition bias and potentially overestimated the intervention effects.

Third, outcomes were assessed only at baseline and immediately after the 8-week intervention. Therefore, the durability of the observed quality-of-life improvements and longer-term intervention effects could not be assessed.

Fourth, the principal outcomes were caregiver-reported and may have been susceptible to reporting bias. Because of the nature of the intervention, participants and researchers involved in intervention delivery could not be blinded, although statistical analyses were conducted by a researcher blinded to group allocation.

Finally, the researcher-developed questionnaire used to collect sociodemographic and caregiving characteristics did not undergo formal psychometric validation.

## Conclusions

In this 8-week randomized controlled trial, a user-centered mobile application developed to address caregiver-identified needs across the dementia trajectory was associated with improvements in overall and psychological quality of life among informal caregivers of PLwD. No significant intervention effects were observed for caregiver burden, neuropsychiatric symptoms of PLwD, or caregiver distress associated with these symptoms. The application also demonstrated good usability among intervention participants who completed the study.

These findings suggest potential benefits of user-centered mobile support for caregiver quality of life. Larger studies with longer follow-up are needed to further evaluate these findings, assess their sustainability, and examine the role of application engagement in intervention outcomes.

## Supplementary Information

Supplementary Material 1.

## Data Availability

The datasets generated and analyzed during the current study are not publicly available due to ethical and privacy restrictions but are available from the corresponding author on reasonable request.

## Abbreviations

MRC: Medical Research Council
ZBI: Zarit Burden Interview
WHOQOL-BREF: World Health Organization Quality of Life Scale Brief Version
NPI: Neuropsychiatric Inventory
SUS: System Usability Scale
CONSORT: Consolidated Standards of Reporting Trials
RCT: Randomized Controlled Trial
SD: Standard Deviation
