# Supplementary material for: Development and effectiveness of a user-centered mobile application for informal caregivers of people living with dementia: randomized controlled trial
Source: BMC Geriatr. 2026 Sep 4;26:1137. doi: 10.1186/s12877-026-08235-7 (PMC13546512; doi:10.1186/s12877-026-08235-7)
Supplement: Supplementary file 1 [file 12877_2026_8235_MOESM1_ESM.pdf]

Supplementary File 1

Representative screenshots of the mobile application interface, key functions, and researcher dashboard

The mobile application was developed and delivered in Turkish for Turkish-speaking informal caregivers of people living with dementia. Therefore, the screenshots are presented in the original interface language. The English figure titles and descriptions below summarize the corresponding interface elements and key functions of the application used during the randomized controlled trial.

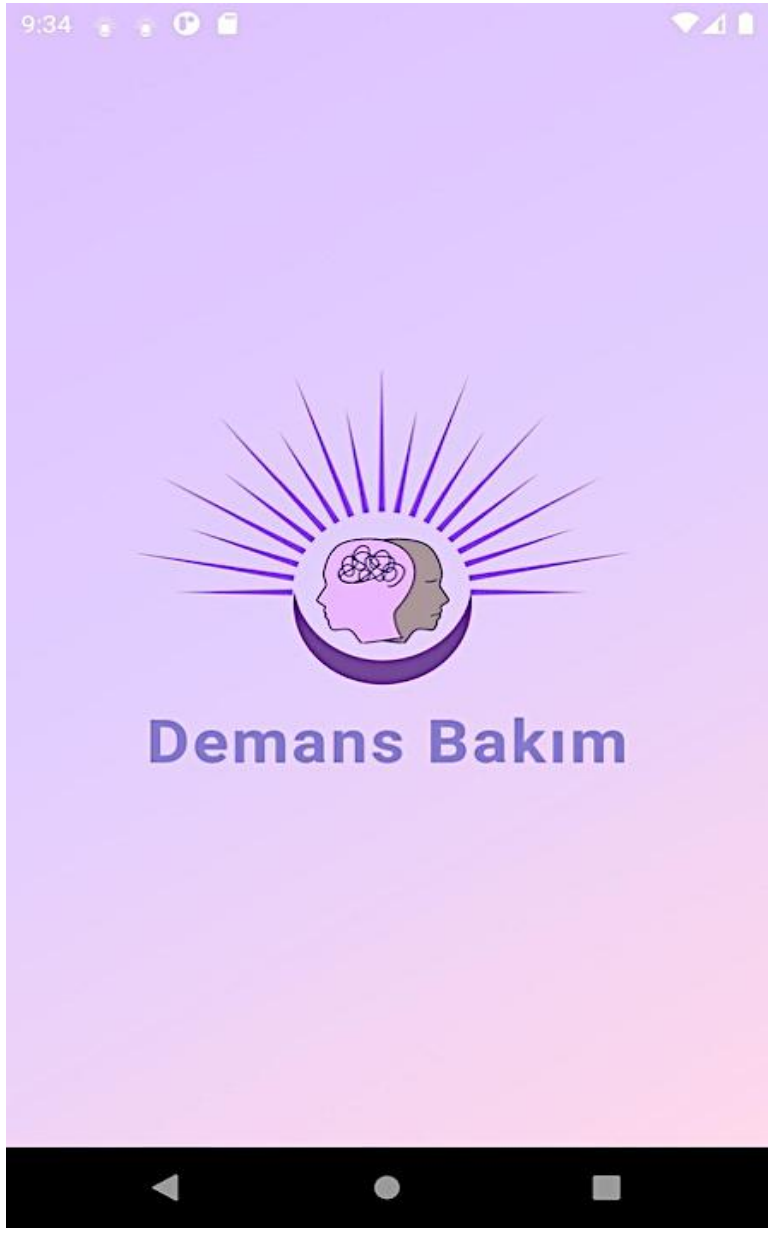

Figure S1. Application name and icon. Opening screen displaying the application name and icon developed for the mobile application.

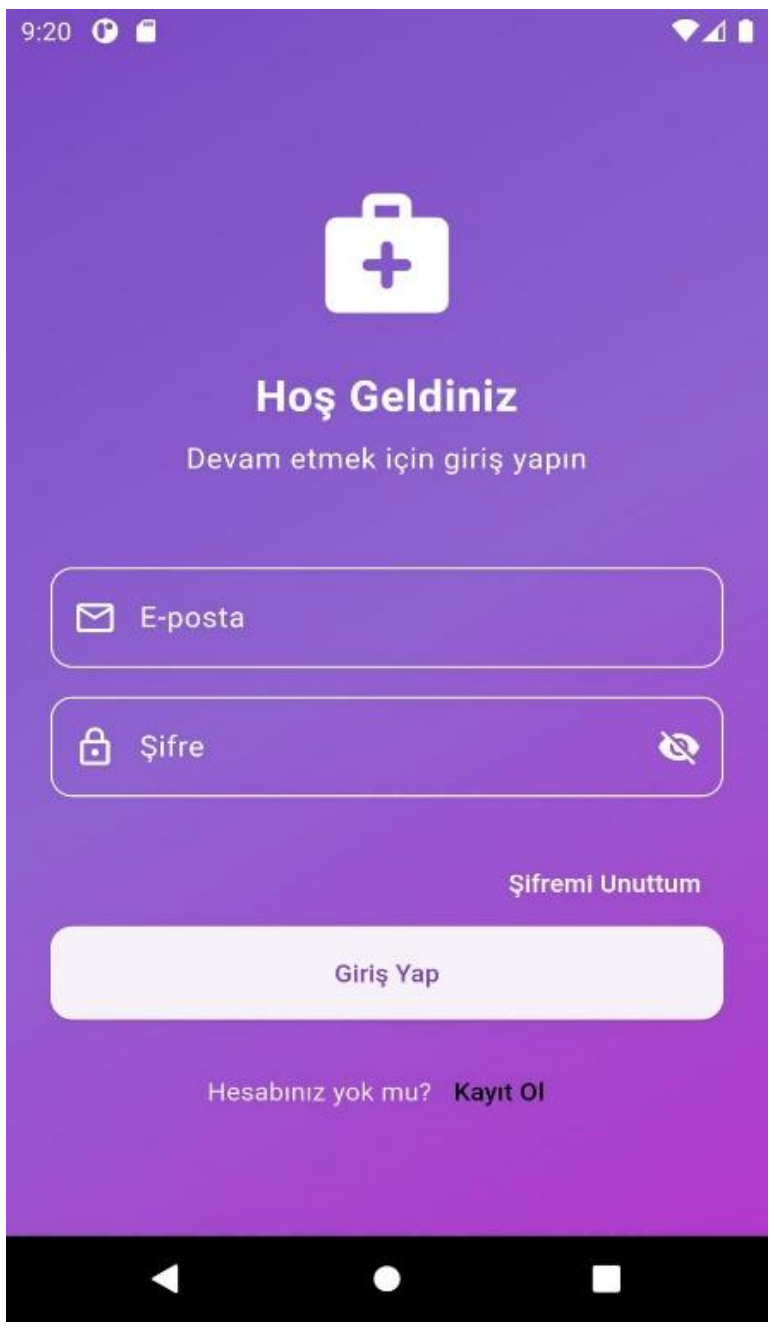

Figure S2. Login screen. The login screen allowed users to access the application using their individual email address and password. A password-reset option was also available for users who had forgotten their password.

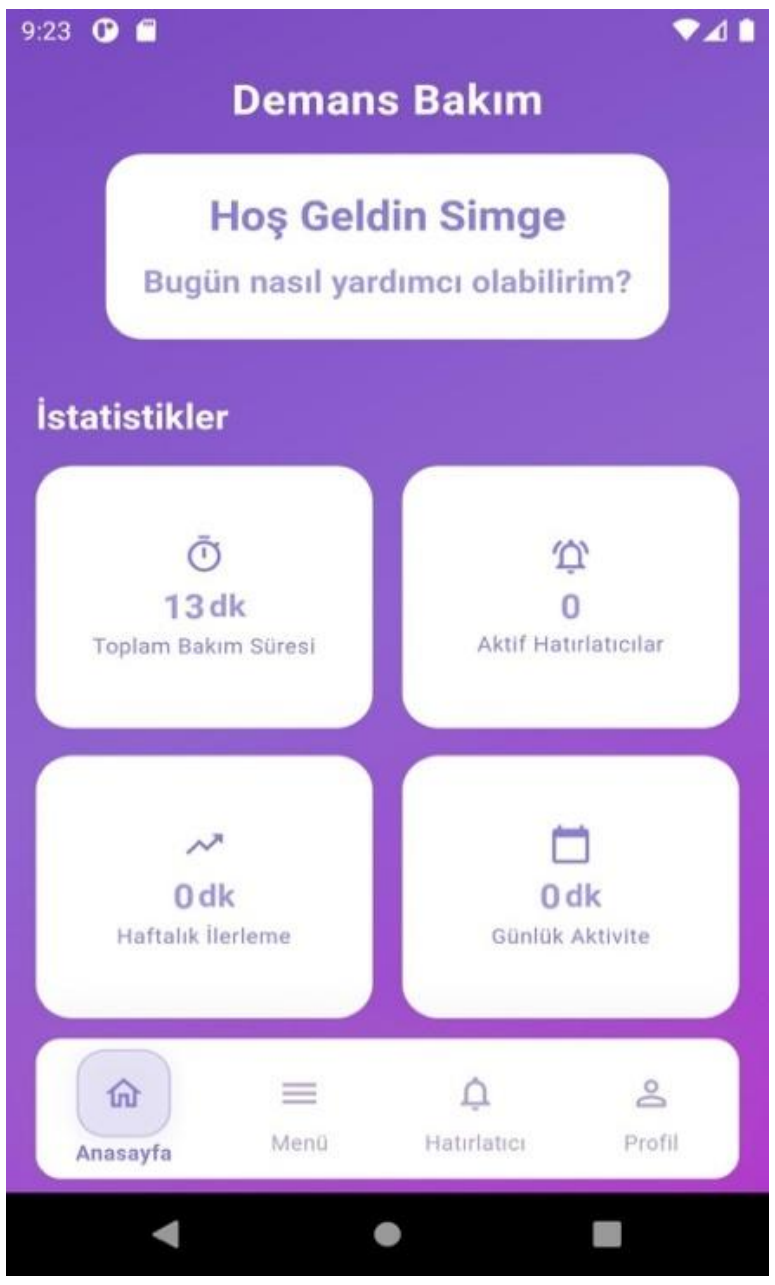

Figure S3. Home screen. The home screen provided direct access to the main menu, reminder, and profile sections. It also displayed current reminders and application-use information, allowing users to view these details from the main screen.

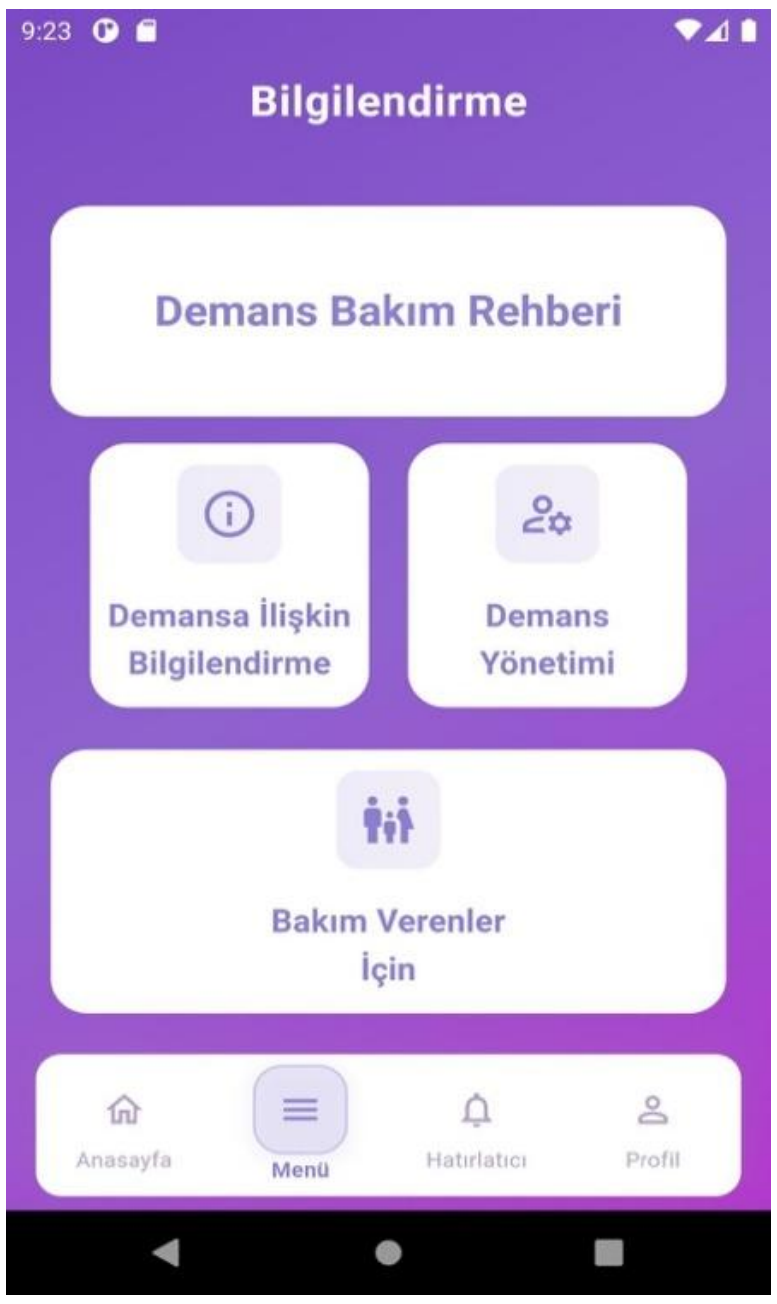

Figure S4. Main menu. The main menu provided access to the application's three main content modules: dementia-related education, dementia management and daily-care support, and caregiver support and self-care.

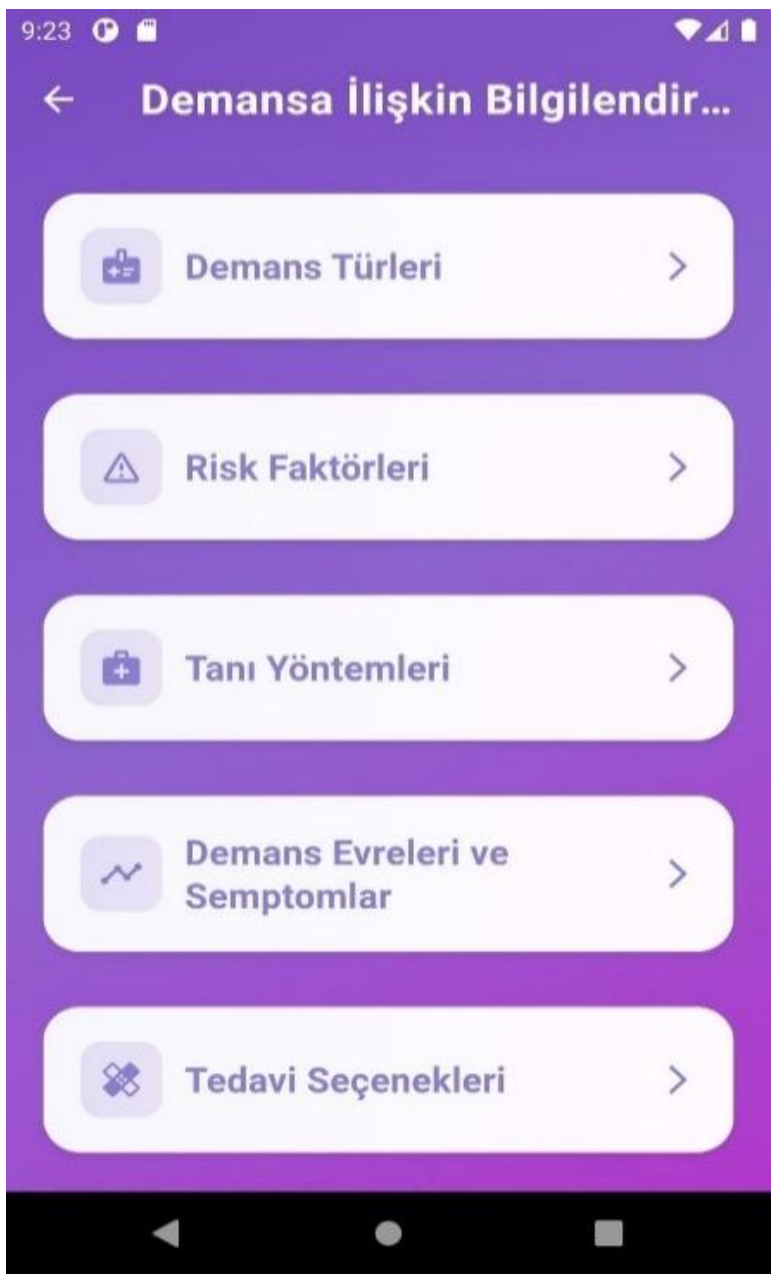

Figure S5. Dementia-related education module. This module provided structured information on dementia, including its definition, types, risk factors, diagnostic approaches, progression and stages, symptoms, and treatment options.

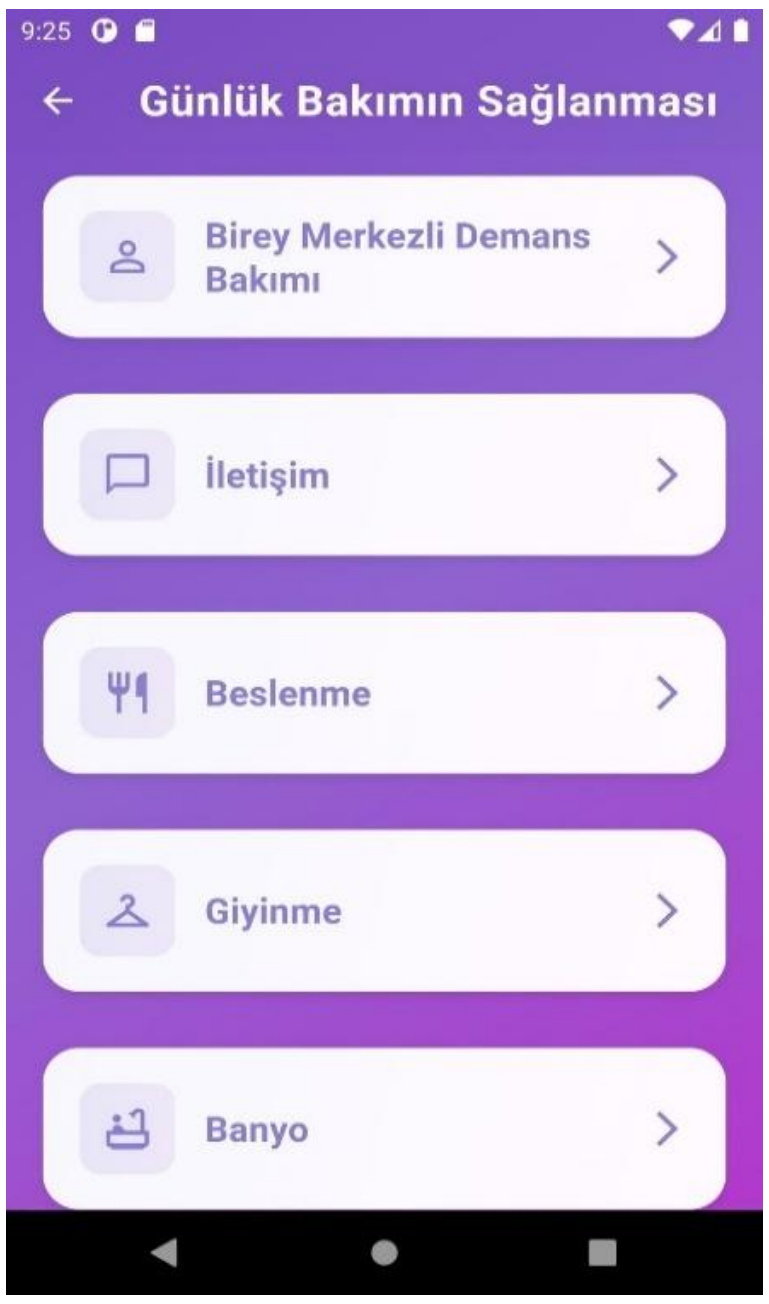

Figure S6. Daily-care support screen. Within the dementia management and daily-care support module, users could access practical guidance on person-centered dementia care, communication, nutrition, dressing, bathing, toileting, mobility and transfers, safety-related issues, shopping, meal preparation, financial activities, and medication management.

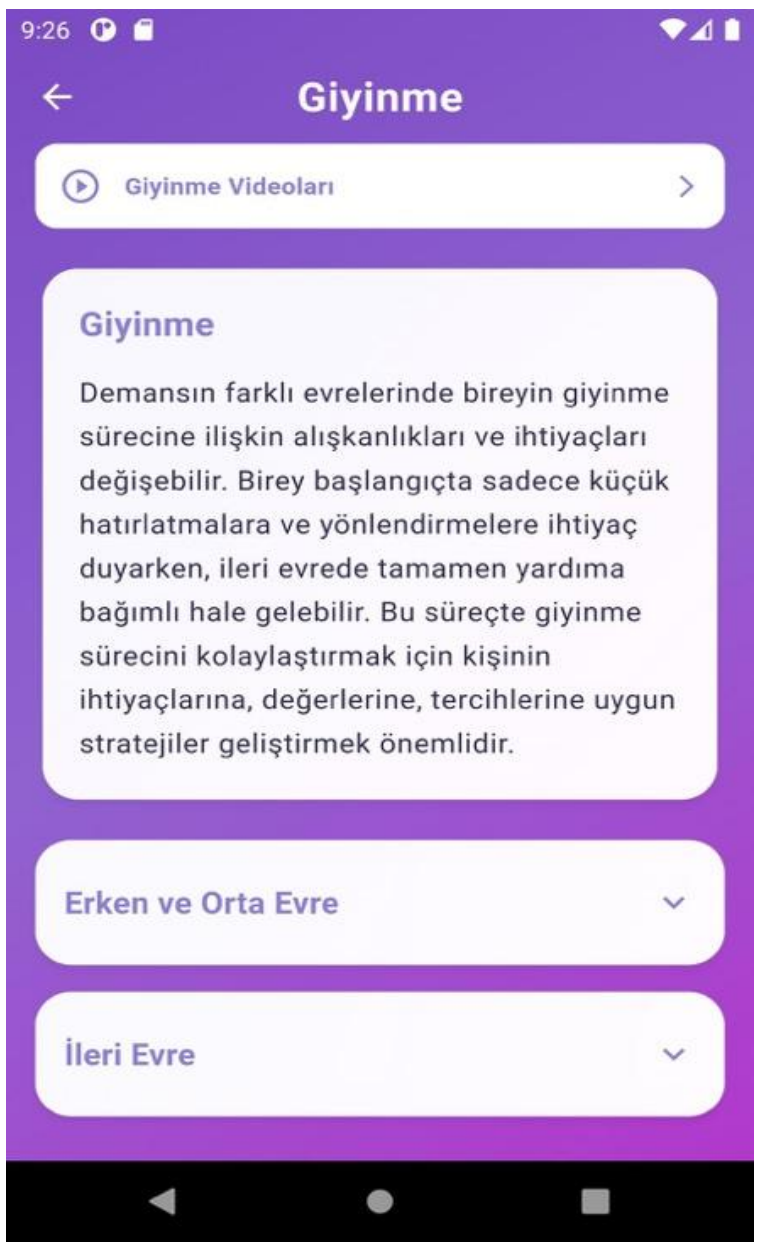

Figure S7. Dressing-care guidance screen. Example of practical daily-care guidance for dressing. Separate early–moderate and advanced sections were available, allowing caregivers to select the content most relevant to the care recipient's current needs.

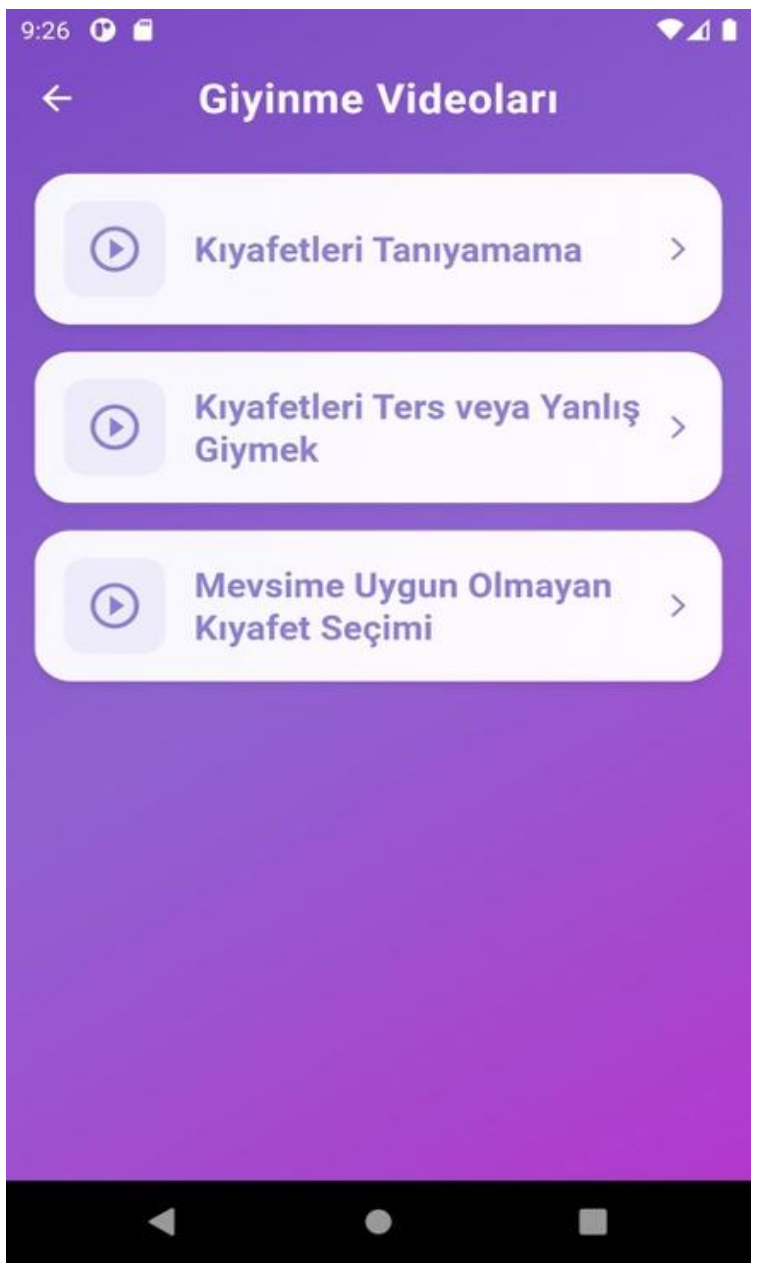

Figure S8. Scenario-based instructional video. Example of a scenario-based instructional video illustrating practical approaches to dressing-related caregiving situations that may arise in daily care.

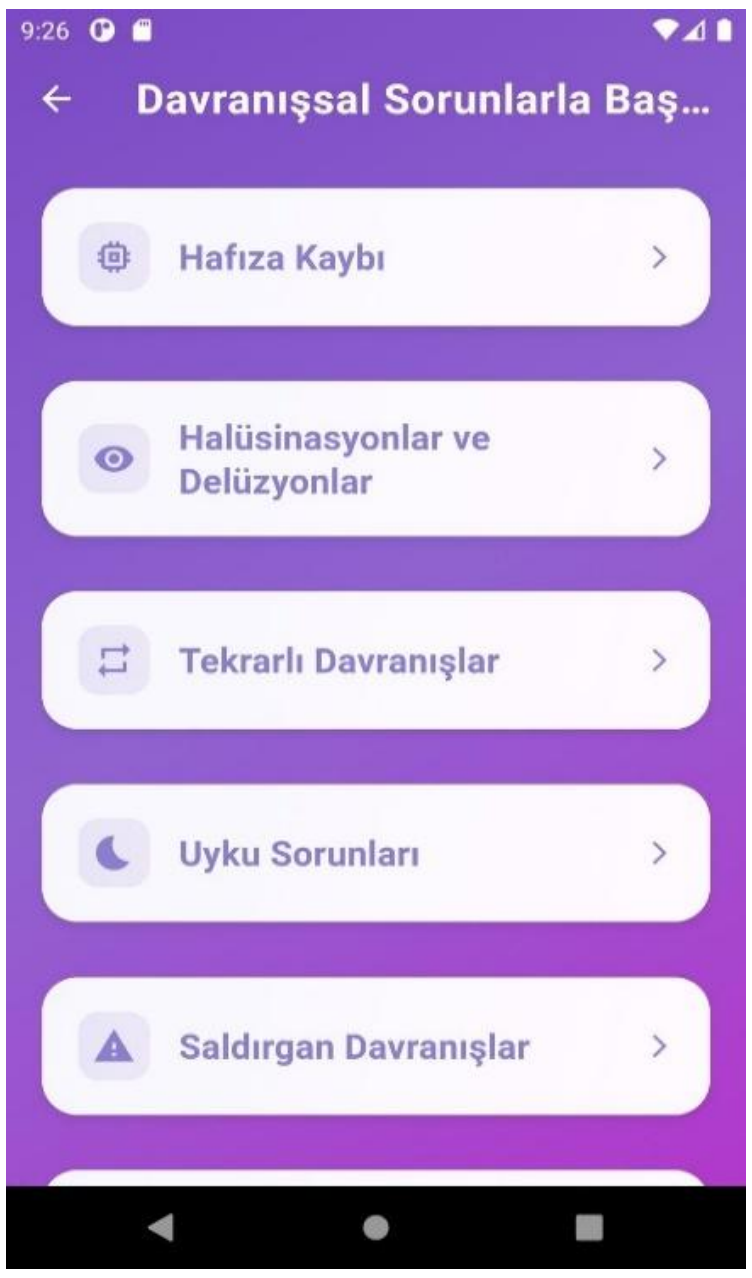

Figure S9. Behavioral and psychological symptom-management screen. This section provided problem-specific guidance on memory problems and common behavioral and psychological symptoms, including hallucinations and delusions, repetitive behaviors, sleep problems, aggression, wandering and getting lost, depression, anxiety, and apathy.

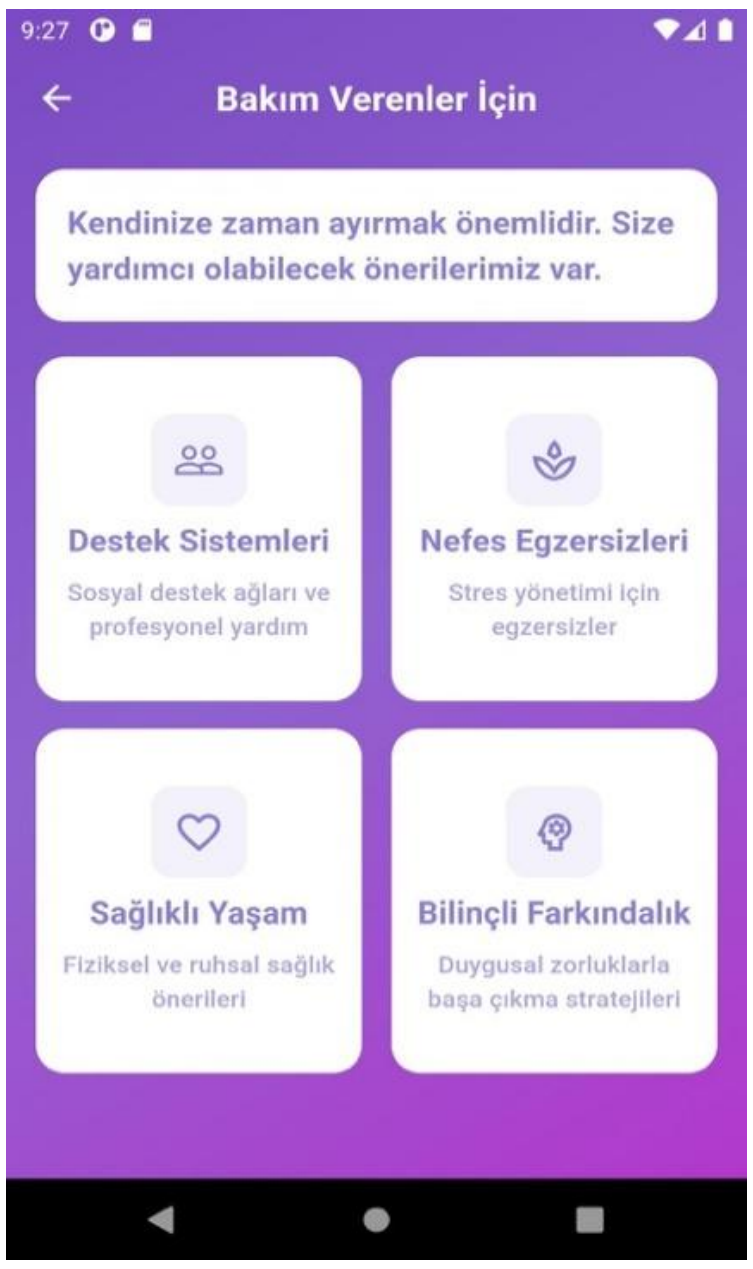

Figure S10. Caregiver support and self-care module. This module provided access to caregiver-focused resources, including support systems, healthy-lifestyle and self-care information, mindfulness practices, and breathing exercises.

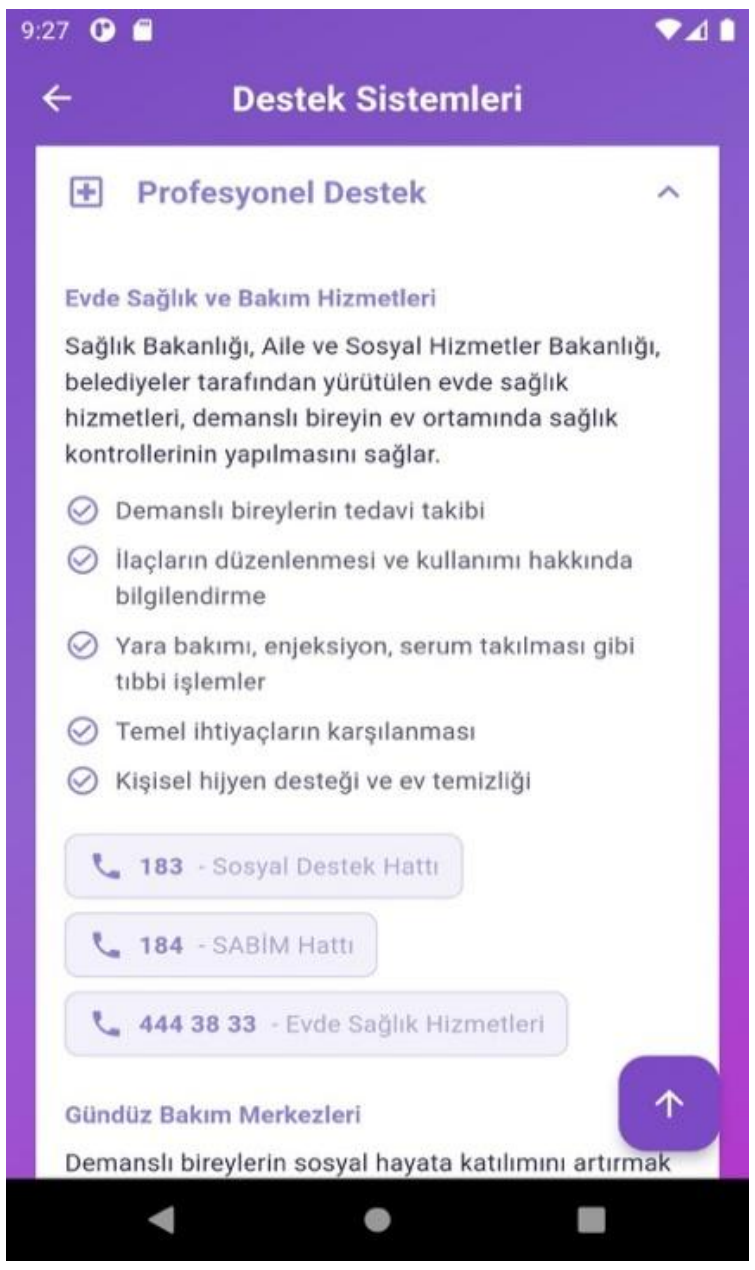

Figure S11. Support systems screen. This section provided information on family and social support, support groups, home health and care services, day-care services, residential care, psychological counseling, crisis-related support, disability-related rights, and financial support.

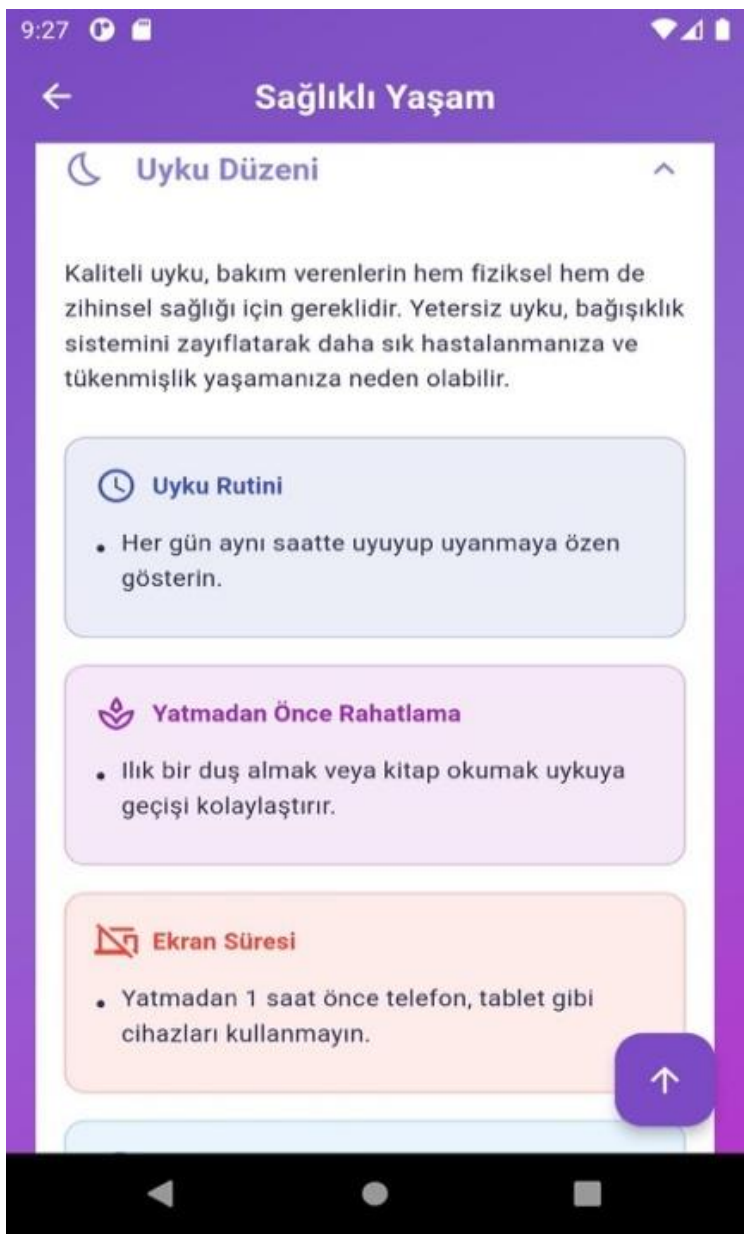

Figure S12. Healthy lifestyle and self-care screen. This section provided information and recommendations on stress management, cognitive reframing, physical activity, nutrition, sleep, hobbies, and personal time.

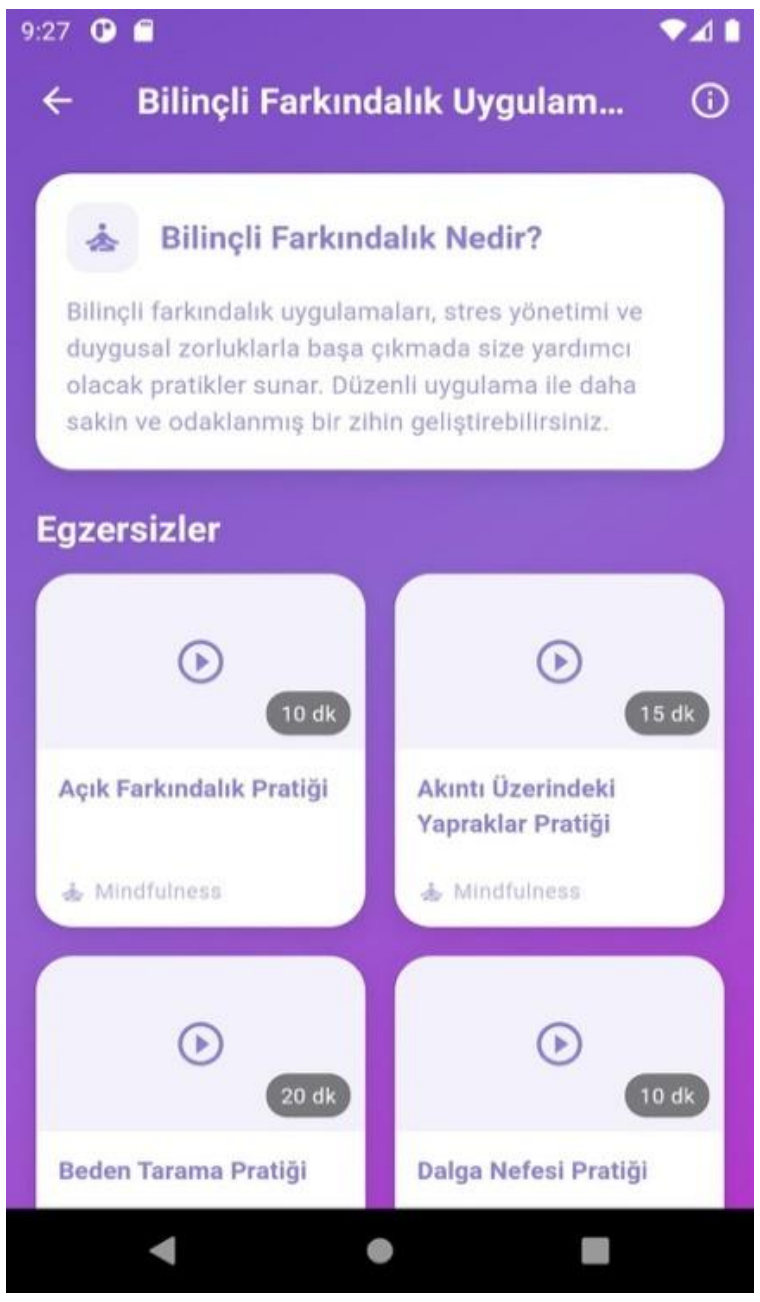

Figure S13. Mindfulness practices screen. This section provided caregiver-focused mindfulness materials delivered through video and/or audio content.

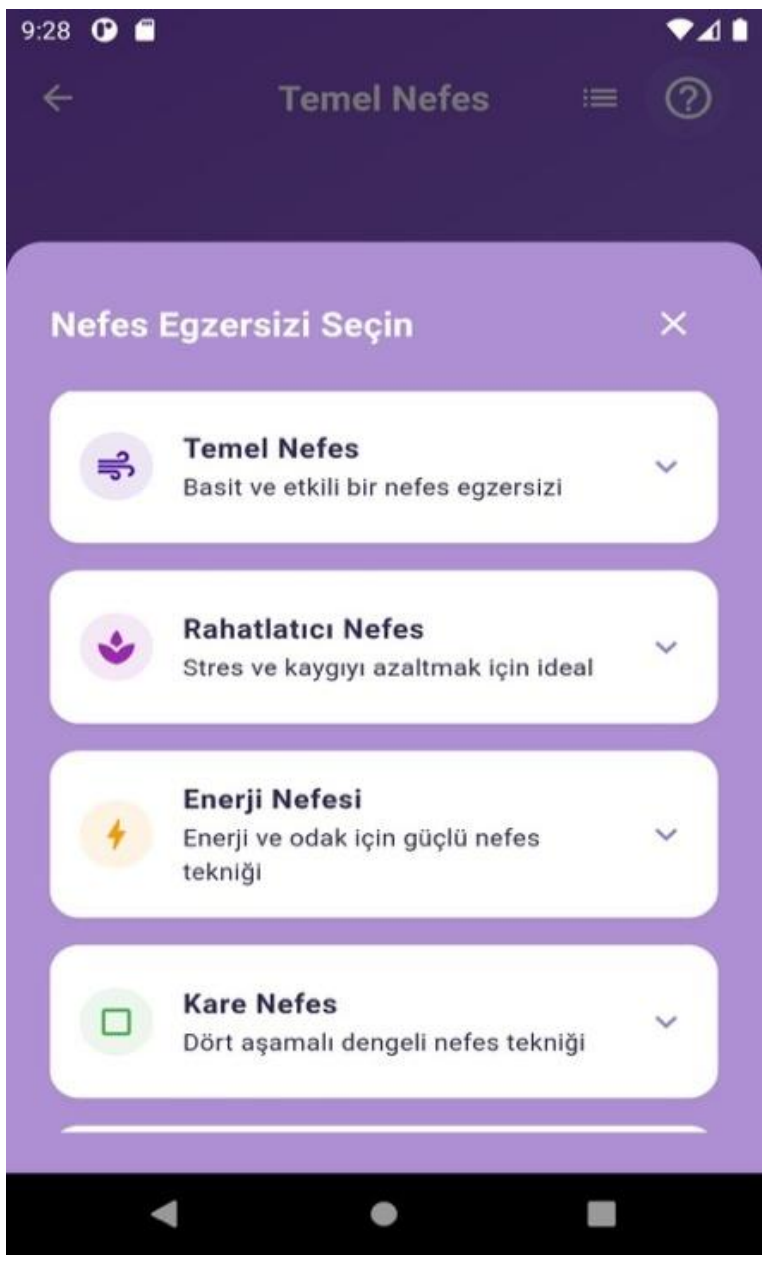

Figure S14. Breathing-exercise screen. This section provided guided breathing-exercise materials through video and/or audio content as part of the caregiver self-care resources.

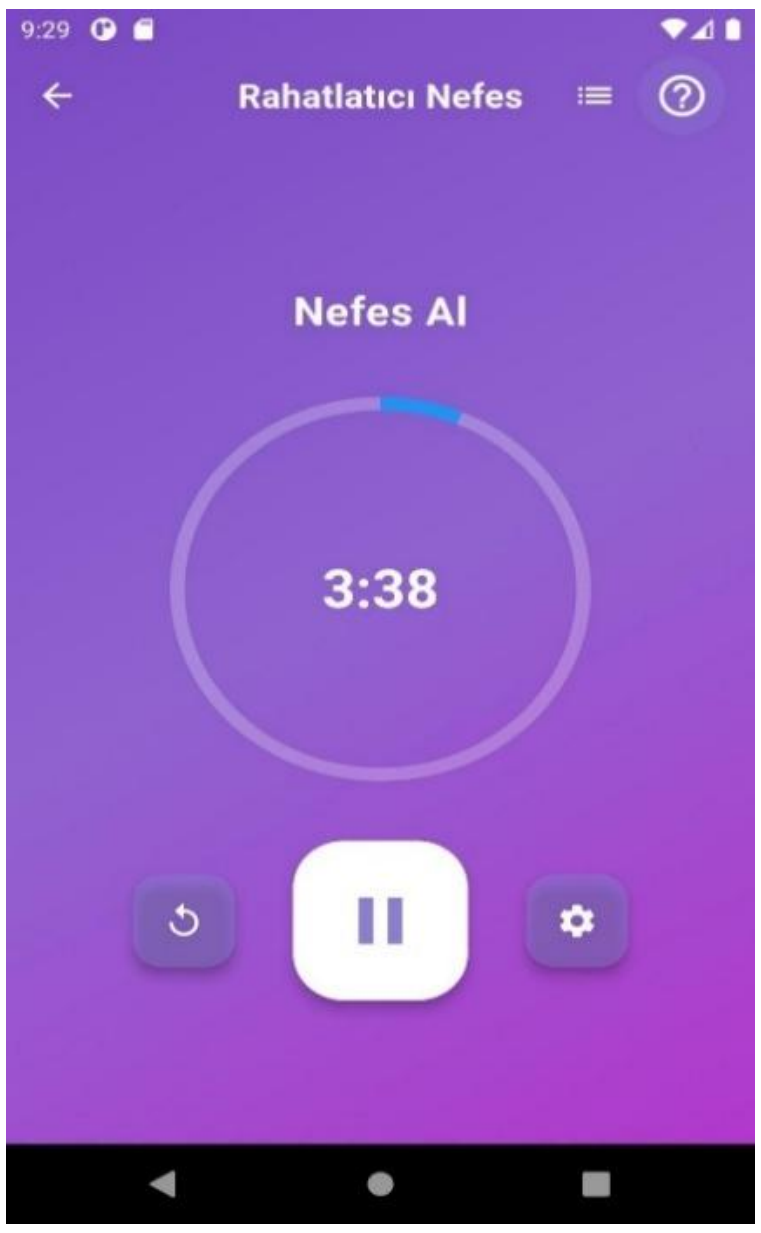

Figure S15. Customizable breathing-exercise function. The breathing-exercise function allowed users to select their preferred inhalation, breath-holding, and exhalation durations. These settings could be saved for subsequent use.

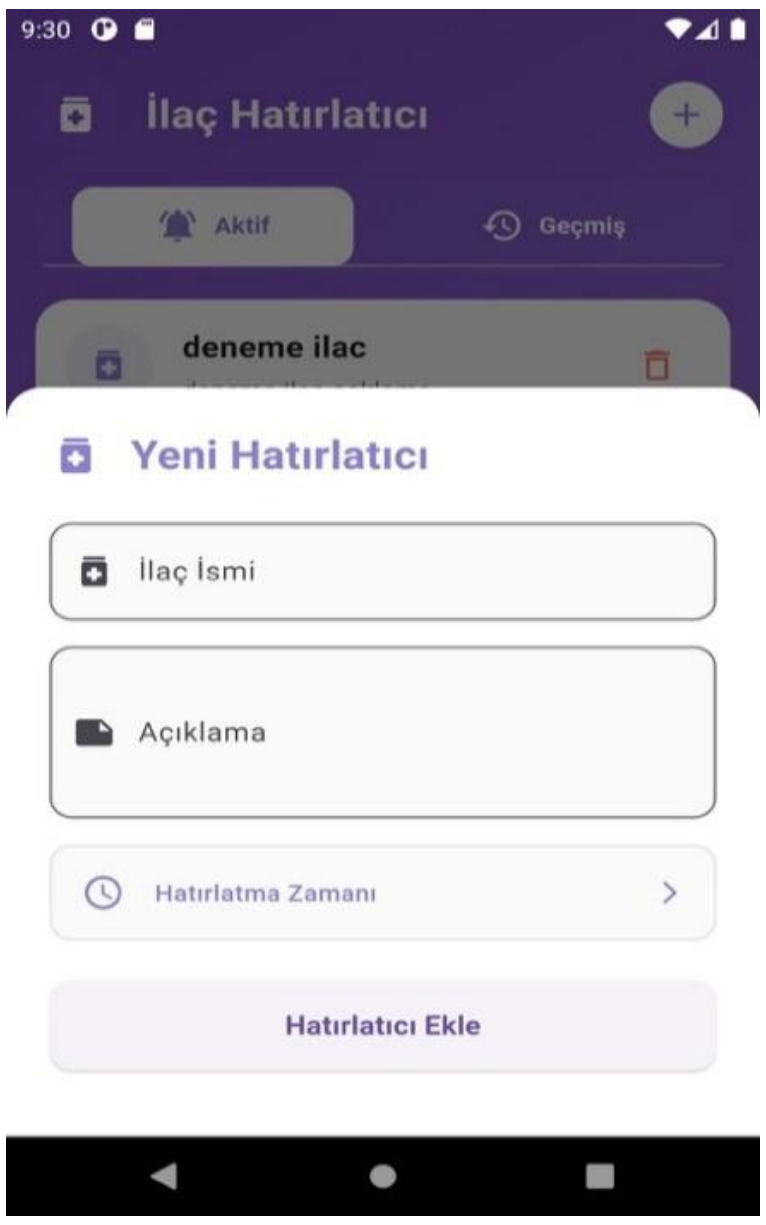

Figure S16. Reminder-creation screen. The reminder function allowed users to create reminders for medications, appointments, toileting, hydration, cognitive activities, walking, and other daily-care activities.

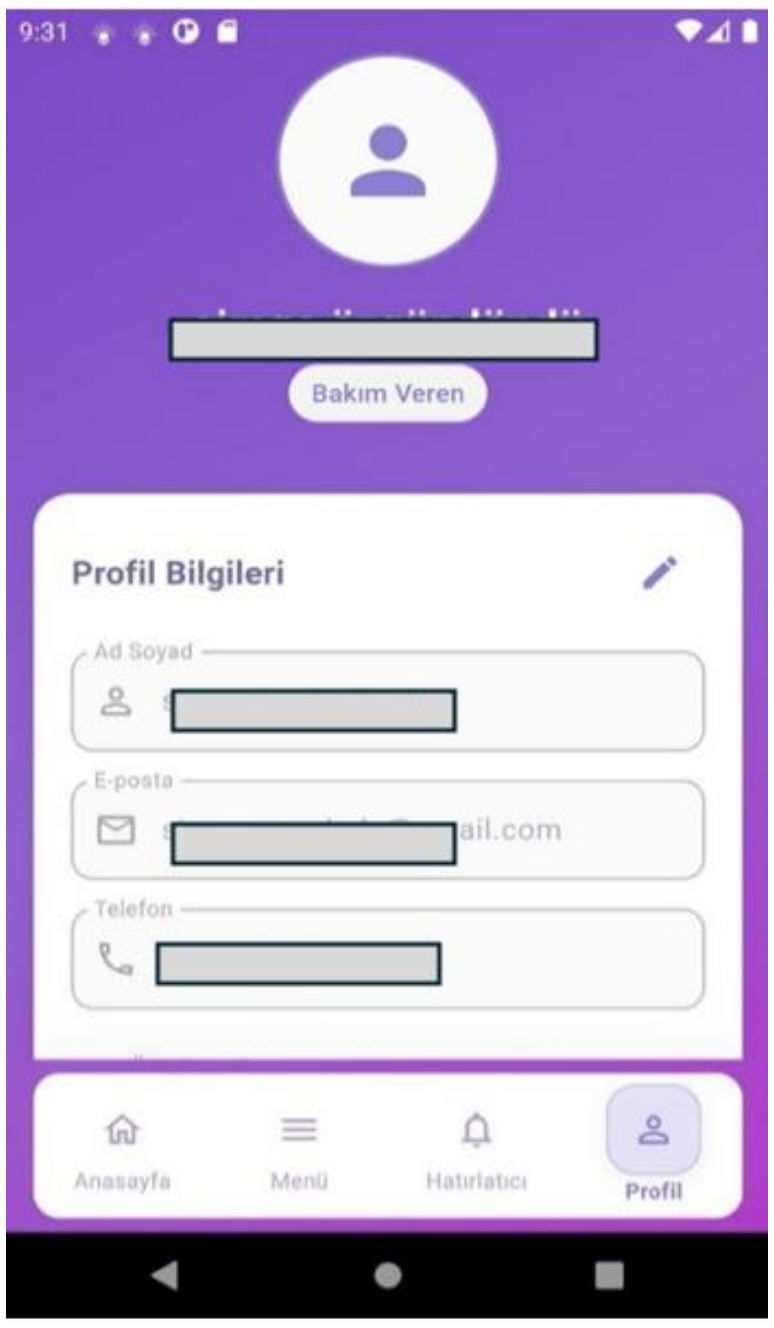

Figure S17. Profile screen. The profile section provided access to users' personal information, privacy and data-security information, password management, and logout functions.

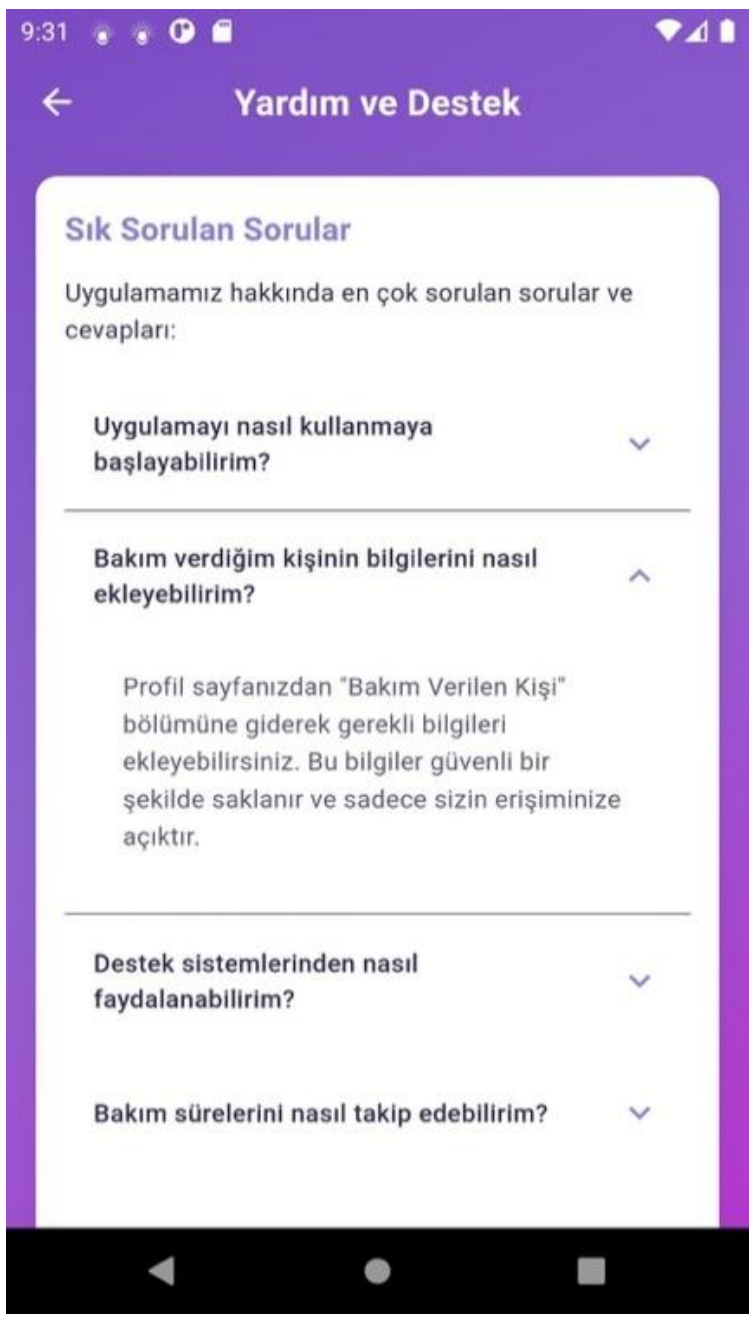

Figure S18. Frequently asked questions screen. The frequently asked questions section provided guidance on common questions related to application use.

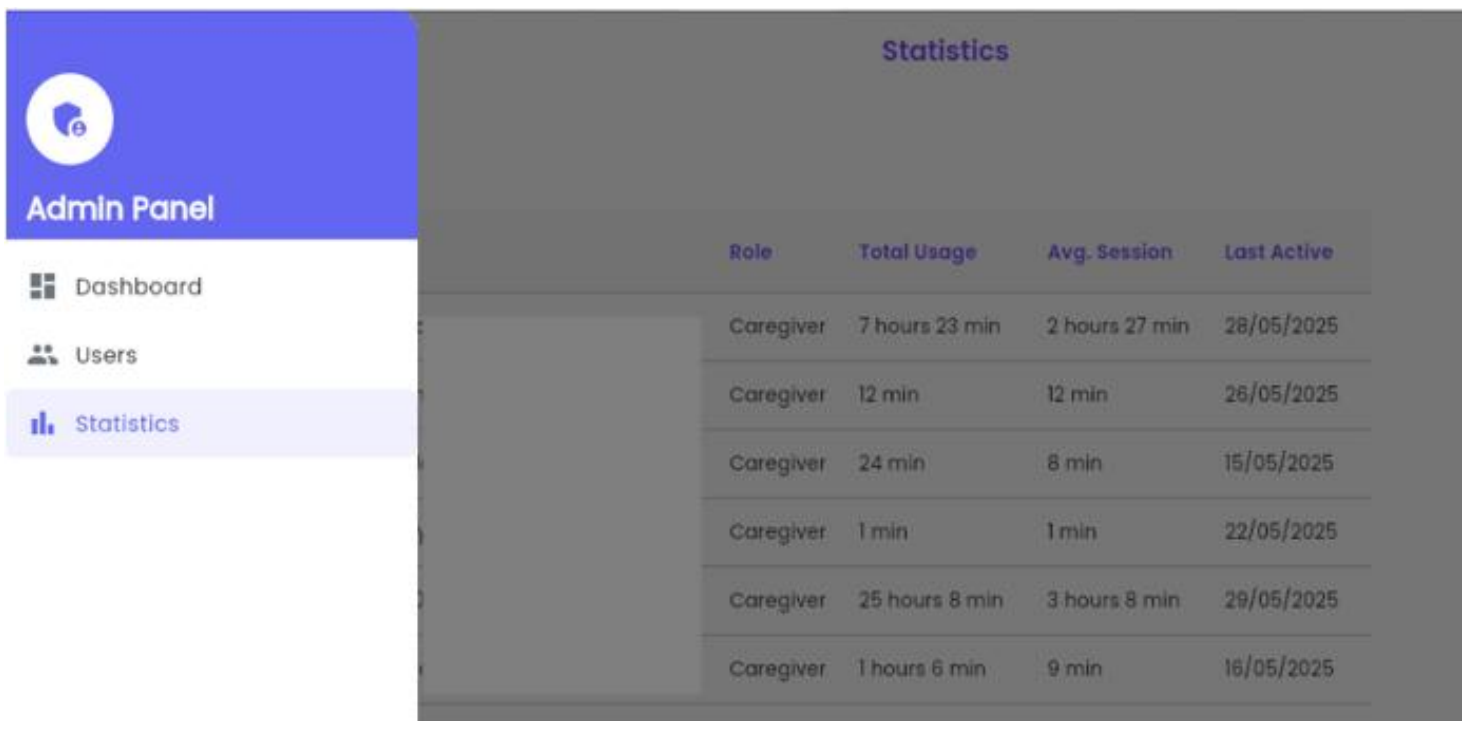

Figure S19. Researcher dashboard. The researcher-facing dashboard was used to monitor application use during the intervention period. It displayed cumulative application use, average session duration, and the date of most recent activity. The dashboard was used for intervention monitoring and was not a caregiver-facing component of the application.
